# Development of the Jackson Heart Study Coordinating Center

**DOI:** 10.3390/ijerph6051597

**Published:** 2009-05-06

**Authors:** Brenda W. Campbell-Jenkins, Clifton C. Addison, Lavon Young, Pramod Anugu, Gregory Wilson, Daniel Sarpong

**Affiliations:** Jackson Heart Study, 350 West Woodrow Wilson Drive, Suite 701, Jackson, MS 39213 USA; E-Mails: Clifton.addison@jsums.edu (C.A.); lavon.young@jsums.edu (L.Y.); Pramod.r.anugu@jsums.edu (P.A.); Gregory.wilson@jsums.edu (G.W.); dsarpong@jsums.edu (D.S.)

**Keywords:** Jackson Heart Study, Coordinating Center, CVD, African Americans

## Abstract

The public health burden caused by cardiovascular disease (CVD) continues to adversely affect individuals in terms of cost, life expectancy, medical, pharmaceutical and hospital care. This burden has been excessive in the case of African Americans. The objective of this paper is to chronicle the procedures and processes that were implemented in the development of the Jackson Heart Study Coordinating Center. The Jackson Heart Study (JHS) is a population-based investigation of traditional and emerging risk factors that predict progression to CVD among African Americans. In response to the struggle against CVD, the Jackson Heart Study has convened a professional, technical, and administrative staff with specific competence in the operation of a coordinating center to handle the wide variety of areas related to CVD studies. The Jackson Heart Study Coordinating Center (JHSCC) was created to assure validity of the JHS findings and provide the resources necessary to meet comprehensive statistical needs (planning, implementing and monitoring data analysis); data management (designing, implementing and managing data collection and quality control), and administrative support. The JHSCC began with a commitment to support study functions in order to increase participant recruitment, retention and safety, meet regulatory requirements, prepare progress reports, and facilitate effective communication with the community and between all JHS centers. The JHSCC facilitates the efforts of the JHS scientists through the development and implementation of the study protocol. The efforts of the JHSCC have resulted in the successful preparation of scientific reports and manuscripts for publication and presentation of study findings and results. In summary, the JHSCC has emerged as an effective research mechanism that serves as the driving force behind the Jackson Heart Study activities.

## 1. Introduction

The Jackson Heart Study (JHS) was designed to be a partnership among three institutions, University of Mississippi Medical Center (overseeing the Exam Center, where participants’ samples, measurements, and data are gathered), Jackson State University (overseeing the Coordinating Center, where the data are managed, stored, and analyzed), and Tougaloo College (operating the Undergraduate Training Center where ethnic minority scholars are provided with opportunities to gain experience in medical/public health research). The JHS is a study of the characteristics of cardiovascular disease (CVD) in African Americans in Mississippi, examining traditional and emerging risk factors that predict progression to clinically detected CVD. The objective of this paper is to chronicle the procedures and processes that were implemented in the development of the Jackson Heart Study Coordinating Center. In recent times, CVD has been documented as a major cause of death in the United States. Approximately 64 million people in the United States have some form of CVD [1]. The public health burden caused by CVD adversely affects finances, life expectancy, and medical, pharmaceutical and hospital care, particularly in the case of African Americans. Since national healthcare expenditures are predicted to reach $2.8 trillion by the year 2011 [2], examining these high risks and the progression of CVD, while monitoring health status, are all important tasks complementary to the improvement of the quality of life for all humanity.

As part of its role in the three institution partnership that comprises the Jackson Heart Study, Jackson State University was entrusted with the task of developing and managing the Jackson Heart Study Coordinating Center (JHSCC). The activities of the JHSCC were designed to provide opportunities for scientists to participate in ventures that would eventually lead to a reduction in the disparities in health care and health services that are now prevalent in Mississippi [3]. Among adults 35–64, the highest rate of premature coronary heart disease occurred in the rural southern region of the United States that is heavily populated by African Americans [4–6]. A 2002 report by the Institute of Medicine demonstrated that racial and ethnic minorities in the United States carry a disproportionate share of the burden of disease and disability [7]. Even though the overall health status of all U.S. racial and ethnic groups has improved steadily over the last century, disparities in major health indicators between white and African-Americans have been growing [8,9]. Adequate data management and data collection procedures had to be developed to obtain consistent, reliable, objective data for this large number of participants [10].

The JHSCC collaborates with the National Heart, Lung, and Blood Institute (NHLBI) that has a field office located on site at the Jackson Medical Mall, Jackson, Mississippi; and the National Center on Minority Health and Health Disparities (NCMHD). The NHLBI Field Site Office team participates in surveillance activities, manuscript development and publication and presentation activities. These activities are fundamental to maintaining the scientific rigor and productivity of the study, and the JHSCC benefits immensely from the continuous involvement of the NHLBI and its Field Center staff.

## 2. Study Procedures

Jackson Heart Study researchers are studying a sample of 5,301 men and women aged 21 years or older who were recruited from the tri-county area of Hinds, Madison and Rankin counties in the Jackson Metropolitan Area. The cohort comprised 3,394 females and 1,907 males. Each participant receives an extensive physical examination and interview to obtain data on coronary status and function, standard coronary risk factors, socio-demographic factors, lifestyle factors, and psychosocial factors. Selected examinations and interview questions are repeated allowing the scientific investigators to comprehensively study the progression of CVD. Blood samples are analyzed and stored for future studies. DNA is extracted and stored for study of candidate genes and possibly, genome-wide scanning. Participants are monitored annually for identification and characterization of CVD events. In order to successfully undertake the enormous task of examining CVD in African Americans, the Jackson Heart Study comprises a professional, technical, and administrative staff with specific competence in the operation of a coordinating center to handle the wide variety of areas related to CVD studies.

### 2.1. Origins and Development of the Jackson Heart Study Coordinating Center

Jackson State University was funded beginning October 15, 1997 to develop plans for the provision of the typical functions of a coordinating center for the Jackson Heart Study (JHS) utilizing The University of North Carolina at Chapel Hill, through its established Coordinating Center, provided consultation to the planning committee. The primary mission was to assure validity of the JHS findings and provide the resources and expertise necessary to meet comprehensive statistical needs (planning, implementing and monitoring data analysis); data management (designing, implementing and managing data collection and quality control), and administrative support. The JHSCC also participated in activities that supported participant recruitment and retention through the Community Partnership Office which maintains collaborative enduring relationships with community groups and leads the efforts to provide community public health education, and to apprise the community periodically of JHS study by delivering progress reports. Several individuals played key roles in the early stages of the JHSCC to plan, develop, maintain, and monitor the effective management of the JHSCC activities. They are the following:

**Robert Garrison, Principal Investigator Coordinating Center,** with responsibility for the overall direction, administration and scientific aspects of the coordinating center, was the representative of the Coordinating Center on the steering committee. He was later replaced by **Daniel Sarpong** in that capacity. In addition, **Dr. Sarpong** participated in policies and procedures development, training, data management activities, cost control, staff management, and report writing, and developed SAS programs for statistical data analysis.

**Mary Lou Gutierrez-Mohamed, Coordinating Center Coordinator,** had primary responsibility for the coordination of the Coordinating Center programmatic activities and participated in the development of the manual of operations, OMB protocols, quality assurance and control, training, data collection, recruitment and retention surveys, and reports development.

**Gail Hughes, Co-Principal Investigator and Assistant Director,** assisted the Principal Investigator in the planning and implementation of all the tasks of the Coordinating Center. She was involved in protocol development issues, sub-studies, ancillary studies, and statistical analysis of data.

**Joseph Cameron, Community Mobilization Director,** provided overall programmatic direction to the Jackson Heart Study Community Mobilization effort, focus group planning and implementation, media outreach and community surveillance. He participated in the recruitment and retention efforts. He was later replaced by **Donna Antoine LaVigne,** and this department was renamed the Community Partnership for Awareness Education with **Dr. Antoine-LaVigne** coordinating the community mobilization-implementation plans, marketing efforts, workshops, conferences, focus groups and public relations.

**Pramod Anugu, Systems Analyst,** provided database system and application support to the study. He designed, implemented, and maintained the systems for regular analysis and dissemination of data; he prepared data-driven reports, maintained and updated the web-sites and the communication systems (LAN etc.).

**Gregory Wilson** served as **Senior SAS Analyst/Programmer,** responsible for preparing the consolidated database, implementing the data management procedures and programming for transfer and import of exam center, laboratory, and reading center data into the consolidated database. He was also responsible for overall statistical computing programming for the study.

**Clifton Addison, Statistical Programmer/Clinic Liaison** participated in statistical computing programming for the study, OMB protocols, quality assurance and control, manuscript development and training. As Clinic Liaison, he was responsible for training and certification of Exam Center staff in preparation for the participants’ exam visits.

**Brenda Campbell-Jenkins, Research Associate** had responsibility for the collection of research materials, preparation of paper forms, question-by-question instructions, and OMB protocols. She served as the Coordinator of the Publications and Presentations Subcommittee and the Ancillary Subcommittee. She also assisted in the QC monitoring of exam clinic through reviewing forms, re-certification documentation, and other standard QC procedures. She maintained detailed and organized records of procedures and served as the repository for all JHS research related information.

**Lavon Young, Data Entry Assistant/Information Processing Specialist,** with responsibility for forms development, preparation of screen layouts for the DMS, and routine data checks, assisted in monitoring retention and follow-up of the cohort through weekly reports in the early stages of the study. She was responsible for formulating, planning and coordinating the layout and typesetting for various documents using advanced software applications.

**Danita Graham, Contracts Administrator,** with primary responsibility for the coordination of the Coordinating Center programmatic activities, as well as the fiscal management of the study, coordinated all purchases for the Jackson Heart Study, communicated with reading centers, sub-contractors, and consultants on all contracts management issues; she tracked expenditures and developed all in-house financial reports for the Coordinating Center, as well as the ancillary administrative budget for Jackson Heart Study.

**Clara Fortner, Senior Program Coordinator,** coordinated, facilitated, and managed activities of the Project Director Office and held overall administrative responsibility for the management of the JHS offices. She supervised all clerical staff and student assistants, developed personnel and steering committee reports, oversaw travel logistics for meetings of staff, sub-contractors, boards, and committees. She also served as the recording secretary of the steering committee.

Planning a study of this magnitude with a partnership among three institutions was an organizational challenge that required an atmosphere of cooperation among the institutions in the planning phase. During this planning phase, the principal activities included designing the survey instruments, developing a sampling plan and examination components; creating manuals of operations and training of staff. It also included pre-testing all data collection forms; implementing procedures for processing data and specimens; performing quality control and data analysis, and obtaining Office of Management and Budget (OMB) study clearance.

### 2.2. Operation of the Jackson Heart Study Coordinating Center (JHSCC)

The JHSCC plays a major role in instrument design, data collection, quality control monitoring, data processing, and statistical analysis in order to document procedures. The leadership at the coordinating center is committed to direct the study through the following sub-committees: Quality Assurance, Publications, Community Mobilization, Operations, Imaging, Laboratory, and Morbidity and Mortality. The JHSCC Director is responsible for maintaining the management structure of the coordinating center and assuring cooperation of all institutional investigators and staff to achieve the goals of the study. He is assisted by a staff of biostatisticians, data analysts, research associates, research assistants, and additional support staff. Participant reports, including summaries of participants’ clinic visits, data quality and timeliness reports, safety reports, inventory information, follow-up status summaries, and reports of unusual problems, are provided by the JHSCC to the entire JHS team.

The JHSCC works in conjunction with the Jackson Heart Study Steering Committee to develop, implement, and monitor procedures to insure confidentiality and privacy in all the data collection stations as well as within the JHSCC. Details about the Jackson Heart Study Steering Committee are chronicled elsewhere. The responsibility for the protection of participants is an integral component of the operational procedures. The JHSCC is committed to ensuring the strictest level of confidentiality by discussing and monitoring procedures during hiring, re-certification, and limiting access, and protecting machine readable data with encryption. The JHSCC plays a major role in protocol development and oversight, instrument design, data collection, quality control monitoring, data processing, and statistical analysis in order to document procedures. Furthermore, it acquires and manages subcontracts for Reading Centers and related activities, cohort recruitment through community mobilization and administrative support.

### 2.3. Protocol Development

The JHSCC facilitates and coordinates the development of the forms, assures uniform implementation of the study protocol and coordinates the maintenance of the Manuals of Operations, providing expert guidance and dependable direction. Manuals of Operations are distributed to all study investigators, and are also located on a study-specific Web Site maintained by the JHSCC. These manuals serve as the main reference for central training of staff, and as a refresher course for staff prior to completing re-certification exercises. The protocols include the Examination Center Operations, Follow-up of health status, and Data and Specimen Collection and Transmittal. The quality assurance and quality control manual includes training and certification requirements, site visit and data audit methods, and procedures for replicate data collection. These documents provide detailed written standard operating procedures that govern the data collection, processing, storage and transmittal, quality assurance, and publication necessary for implementation of the scientific objectives and methods of the study protocols.

The JHSCC guides the development, integration, and production of all Manuals of Operation, under the direction of the Steering Committee, including a detailed set of question-by-question instructions (QXQs) for each of the interview instruments for the target groups. Protocol development, staff training, and pilot testing were performed in the first 18 months of the study. The first examination took place over a four year period, from September 2000 to April 2004. It was followed by a second examination period beginning in September 2005 that ended in December 2008. A third examination period began in February 2009. Participants are contacted every 9 to 12 months throughout the study to assess clinical morbidity and mortality. Data analysis began in the last few months of 2004, and this focus will continue for the rest of the study and beyond.

### 2.4. Data Management, Quality Assurance, Analysis and Reporting

A database management program was developed with an overriding emphasis on ensuring system efficiency and system security. The JHSCC used a uniform approach to security across computer systems and databases. The guidelines required that proper authorization be identified as the first step in the creation of an account. Also included, as part of the security process, was the development of a standard convention for usernames and passwords. In addition, specific procedures were outlined and documented for JHS data management and data protection [11].

During the planning phase of the JHS, systems were developed for examination data, administrative data, follow up data, and the event or surveillance data. Comprehensive data management is provided throughout all phases of data collection. A highly skilled team is on staff to design and develop computer applications for data collection, and to execute plans for central data acquisition, management, analysis, quality control measures, the recruitment and retention tracking system, and surveillance protocols. These staff members also possess expertise in the methods used to coordinate and manage this complex epidemiological study which include recruitment, clinic visits, networks, laboratories, reading centers, and follow-up of participants. Their responsibilities also include assisting in organizing protocol and procedures and monitoring recruitment and follow-up of study participants.

Although the JHSCC started out using paper data collection systems, the transition was soon made to a commercial data management system that was customized over the first two years of operation. This completely electronic data entry and data management system uses commercial customized software or commercial database management system (DBMS) such as Oracle, Oracle Clinical or Clintrial4 and applications developed by the systems analysts. A tested data backup system has been integrated and regular backups are done. Each PC is connected to a central server for administrative and communications functions with standardized, secure e-mail and scheduling software for both the JHSCC and the Exam Center (EC). Communication between the Exam Center (EC) and JHSCC can be done using e-mail and other electronic means. Paper communication and FAX communication are also available means of communication. A secure dial-up capability is provided as a backup to direct internet connection for authorized JHSCC and EC personnel, and all systems are protected from power failure by means of uninterruptible power supplies.

The JHSCC supports a Jackson Heart Study public web site and maintains secure web and file transfer protocol (ftp) sites for controlled information transfer and releases of data residing on the fast, high volume server. The JHSCC also maintains an effective intranet through the use of the secure web pages and secure ftp sites. The public web site publishes general news and technical information approved for public use.

The JHSCC has developed documentation and training programs for all data collection software, instruments, and forms used by the Exam Center (EC) personnel. The system includes procedures for regular sampling inspection of intermediate and final data products for comparison to source information, and data correction and feedback to the EC and reading centers, when data quality problems are encountered. When data are being entered and electronically sent to the database, the staff conducts range checking and database consistency checking, and arranges site visits to observe data collection quality. As a contingency plan, backup systems using paper forms are provided in situations where computer system failures interrupt data collection. This system also includes an analysis management protocol with procedures for tracking data analysis requests, archiving analysis results and programs and error checking and verification of analyses. Data quality is optimized by the data entry, management and reporting system that is resistant to data error and data loss.

### 2.5. Budgeting, Contracting, Purchasing and Maintaining Equipment; Reading Center Operations

An important function of the JHSCC is the evaluation of the suitability of medical and scientific expertise in cardiovascular medicine and the management of contracts and contractual arrangements with consultants specializing in analysis and interpretation of special JHS investigations. The JHSCC works with the NHLBI staff and the examination center to establish the framework for the subcontracts, the selection criteria, and solicitations, in accordance with NHLBI guidelines. This JHSCC oversees all budgets, in addition to contracts with the central processing unit for supplies at Jackson State University, a Central Laboratory, and Reading Centers including carotid ultrasound, electrocardiography (ECG), echocardiogram (ECHO), pulmonary function, diet and other special core center operations. A detailed work plan for each subcontractor is compiled indicating how each aspect of the work is to be accomplished. The JHSCC maintains information on how each sub-contractor’s project is to be organized and staffed, along with the management of important events or tasks with the objective of monitoring their operations to ensure that their proposed scope of work is completed in a timely and cost-effective manner. The details of the reading centers operations are chronicled elsewhere [12].

The JHSCC purchases, distributes, and maintains all mechanical and electronic equipment related to computer hardware and software and clinic furnishings that can not be provided by the JHS centers. Such hardware includes workstations, file servers, laser printers and network components, such as wiring, hubs and connectors. The laboratories and reading centers will receive a stand-alone workstation on which to run the inventory and, if necessary, a data management system. The JHSCC is also responsible for developing, in conjunction with the laboratories and reading centers, a schedule for calibration and maintenance of the equipment. The JHSCC assembles and monitors the information on equipment calibration and maintenance for inclusion in the quality assurance reports.

### 2.6. Community Partnership/Mobilization Activities Addressing Participant Retention/Attrition

Staff of the JHSCC created a community mobilization plan to develop partnerships within the community and enhance methods for fostering recruitment and cohort retention in the JHS. Community mobilization, though not a traditional function of a coordinating center, was helpful in facilitating access to a stable population of African Americans from which the cohort was derived. The community mobilization arm of the JHSCC is titled the “Community Partnership for Awareness Education”. This component makes JHSCC unique compared to other Data Coordinating Centers. Among its main functions are addressing factors that may influence people to participate in long-term research studies, promoting ongoing community support for the Jackson Heart Study, and disseminating information about CVD risk factors to the community. The community mobilization plan undergoes periodic modification to adapt to specific needs of the study and the community, and much of this is executed through a comprehensive community involvement plan that includes the development and implementation of an extensive media campaign. The Community Partnership Subcommittee functions as the oversight advisory committee for this division of the JHSCC and is composed of JHS staff along with representation from the community. This subcommittee meets regularly to monitor progress of the JHS mobilization activities, evaluate JHS directions and achievements regarding recruitment/retention, and to make recommendations for improvements. Barriers and facilitators to African Americans’ participation in research were documented by the planning committee prior to implementation of the JHS.

Knowledge about older African Americans and their involvement and influence in recruitment are essential to understanding recruitment success and pitfalls in ethnic minority research [13]. Most African Americans have very serious concerns about participating in large research studies, and many of these concerns center around attitudes about fairness, protection from harm and understanding of research as a key to improving health care. The JHSCC accentuated the importance of addressing the trust barriers to research participation and the importance of conveying a sense of caring for the health of individuals and the community as a whole as proposed by [14]. The JHS Community Partnership office developed successful strategies in working with African Americans in research by emphasizing local access, local relevance, and decision-making [15]. Activities had to be implemented to enhance retention among the African American population, providing information to the participants and their relatives, and serving as links between the participant and other community resources [16], especially since a significant barrier to participation in research is mistrust of the scientific community and institutions [17].

### 2.7. Administrative Support

The Coordinating Center provides the scientific leadership committees with accurate and timely reports on study progress. Performance reports, including participant accrual summaries, data quality and timeliness reports, safety reports, inventory information, follow-up status summaries, and reports of unusual problems, are provided to all JHS components. The Coordinating Center provides administrative support for the meetings of the JHS Steering Committee and all JHS subcommittees, and conference calls. Annual meetings of the NHLBI/JHS Observational Study Monitoring Board (OSMB) are arranged and managed as part of the administrative support provided by the JHSCC. The Coordinating Center also provides audiovisual equipment, and makes special arrangements to facilitate the successful culmination of proceedings.

### 2.8. Research Support

The Research Support Unit is responsible for providing technical support, as well as training and support for the rest of the JHS departments. Because of the overwhelming nature of research, the Research Support Unit was established to facilitate accessibility, problem solving and project development for beginning researchers and seasoned professionals alike. These staff members are dedicated to assisting interested individuals or groups with understanding and implementing Jackson Heart Study protocol and procedures, and meeting deadlines for deliverables. This unit’s primary goal is to support and assist policy and practice. The Research Support Unit of the JHSCC also facilitates entry and editing of data from the examination site and reading centers; follows up on missing or erroneous data; does quality control monitoring through data checks and monitoring visits; prepares reports on study progress and coordinates and monitors interim contact with study participants by the examination center.

Several mechanisms have been developed to monitor quality control. The JHSCC provides documentation, training and user support along with regular assessment by observing exams and interviews in which direct entry is taking place. The JHSCC establishes staff training and certification procedures, internal quality control programs and data quality procedures. A schedule has been developed for periodic re-certification, calibration of equipment, and pretest of procedures. Protocols have been developed to monitor through direct observation (over-the-shoulder, audio taping), quantitative monitoring (random repeat measurement of inter- and intra-staff), reporting results (timeliness, clarity), and action on results (praises and remedial action).

The JHSCC is designated as the repository of all JHS data; responsible for preparation and dissemination of QC reports using tabulated data, summary statistics, and identifying specific QC problems. The JHSCC developed the Quality Control Protocol, and provides operational support for the quality control committee, through the maintenance of central logs of data quality problems and solutions. Data quality is assured by providing training and pre-testing, performance monitoring, quality control and online documentation. Quality Control is an ongoing monitoring process to detect deviations from protocol standards and procedures. Such monitoring activities take place at identified points during data collection and processing.

### 2.9. Biostatistical Computing-Understanding Scientific Issues and Publishing Study Findings

The JHSCC has responsibility for developing and implementing pre-testing procedures. These procedures are developed for each component of data collection and field tested, and a detailed evaluation is provided to the Steering Committee (SC). The Biostatistics Unit possesses vast knowledge and understanding of scientific issues related to the study rationale and design, including the importance of the research questions and hypotheses, knowledge of CVD, and understanding of state-of-the-art analysis methods. Statisticians in the Biostatistics department possess expertise related to study design and analytical methods. This group takes the leadership in preparation of scientific reports and manuscripts for peer-reviewed publication. The JHSCC Biostatics Unit provides statistical analytical support for all JHS investigators and collaborators at other institutions.

## 3. Conclusions

### 3.1. Acquired Expertise in Managing Large Epidemiological Studies

The investigators and staff of the JHSCC have demonstrated the capacity and the personal and technical expertise to design, organize, and efficiently execute data management and analysis and related responsibilities for a large-scale epidemiological study. Jackson State University has succeeded in establishing a functioning Coordinating Center capable of carrying out the epidemiological, biostatistical, data base management and data storage center resource functions for the Jackson Heart Study (JHS). The JHSCC has demonstrated the leadership and ability to coordinate statistical, medical, data management, and the fiscal/administrative expertise to provide leadership for the operation of a first-class Coordinating Center. The JHSCC continues to meet some very important objectives; to assist medical professionals in their research, understanding, analyses and dissemination of CVD discoveries; to report findings and circulate information to the community in an appropriate, relevant and culturally sensitive manner with the ultimate goal of improving the quality of life.

The development and implementation of the JHSCC has led to the opening of several opportunities for public health students to engage in hands-on research training. The Jackson Heart Study research training opportunities include research projects that can lead to Masters theses or doctoral dissertations. In addition, the JHSCC regularly engages students from local and national colleges/universities in research assistantships and summer internships to prepare them for careers in the medical and public health fields. Through the development and successful implementation of the JHSCC, the Jackson Heart Study continually seeks to fulfill its obligation for capacity building in the African-American community.
